# Feeling the music: The feel and sound of songs attenuate pain

**DOI:** 10.1177/20494637221097786

**Published:** 2022-05-03

**Authors:** Dhillon Lad, Alex Wilkins, Emma Johnstone, Quoc C Vuong

**Affiliations:** 1 School of Psychology, 5994 Newcastle University, Newcastle Upon Tyne, UK; 2 Biosciences Institute, 5994 Newcastle University, Newcastle Upon Tyne, UK

**Keywords:** Pain, music induced analgesia, touch induced analgesia, affect, emotion

## Abstract

**Background:**

Extensive research has demonstrated that music and touch can separately attenuate perceived pain intensity. However, little research has investigated how auditory and tactile stimulation can synergistically enhance pain attenuation by music. In the current study, we investigated whether tactile stimulation can enhance music-induced analgesia for noxious force stimulation on the fingertip.

**Methods:**

We systematically applied force to 34 listeners’ fingertips to induce pain. We then compared the force measurement (in Newton) that gave rise to the *same* perceived moderate pain intensity when listeners were presented their self-selected *liked* or *disliked* song with *auditory-only*, *tactile-only* and *auditory-tactile* stimulation. Higher force indicated less perceived pain. The tactile stimulation were low-frequency modulations extracted from the songs and presented as vibrations on the wrist.

**Results:**

The results showed a significant interaction between song preference and stimulation condition. Listeners had higher force measurements at the same moderate pain for their *liked* compared to *disliked* song only in the *auditory-tactile* condition. They also had higher force measurements for their *liked* song with *auditory-tactile* stimulation compared to the other remaining conditions except for the *liked* song with *auditory-only* stimulation.

**Conclusions:**

The addition of tactile stimulation enhanced music-induced analgesia which reduced subjective pain intensity. The findings suggest that combined auditory and tactile stimulation may increase the affective content of self-selected preferred music, which may stimulate affective and motivation mechanisms which inhibit pain transmission.

## Introduction

Pain perception is subjective. The *same* level of noxious stimulation can induce *different* levels of perceived pain intensity depending on people’s current physiological, emotional and cognitive state.^1–4^ Music is an effective inducer of emotions,^5^ with evidence that listening to music can attenuate acute^6^ and chronic pain.^7,8^ Several factors can affect the magnitude of music-induced analgesia (MIA), including the music’s affective content and whether listeners select their preferred music.^9^

One less-studied factor is how other senses contribute to MIA. The sense of touch is particularly important in this regard for several reasons. Firstly, pain can be attenuated by activation of non-noxious skin receptors.^10^ Secondly, touch and pain signals are processed in the somatosensory cortex.^11^ Lastly since listeners can *feel* the low-frequency vibrations associated with the music’s acoustic energy emitted by speakers (for example) on their skin, tactile stimulation is often used in affective computing to enhance emotions elicited by music.^12,13^ Here we investigated whether combining auditory and tactile (touch) stimulation by music can enhance pain attenuation.

MIA has been demonstrated in listeners exposed to experimental pain.^14–18^ These studies highlight the importance of the music’s affective content in modulating pain via the affective and motivation systems.^19^ For example Roy et al.^20^ found that pleasant music attenuated thermal pain more compared to unpleasant music. Music preference is another factor contributing to MIA.^21,22^ For example Perlini and Viita^6^ showed that participants who listened to their most preferred music reported greater pain attenuation to finger-pressure pain compared to those who listened to their least preferred music. Similarly, Finlay and Anil^23^ demonstrated that self-selected happy music attenuated pain more than self-selected sad music in a cold-pressor test. Overall, the literature suggests that self-selecting music can enhance pain attenuation as listening to preferred music can release dopamine and endogenous opioids.^9^

Touch is important for both pain and music. Tactile stimulation is often applied as vibrations to different parts of the body. Vibrations can be characterised by their frequency and amplitude (intensity). They are similar to sound waves, but are carried on a medium like the skin, instead of the air. Vibrotactile stimulation (vibrations applied to the skin) can activate Meissner’s and Pacinian corpuscles,^24–26^ which are mechanoreceptors in the skin. According to a touch-pain model,^11^ regions of the primary somatosensory cortex respond to both tactile and noxious signals. When these signals are at a moderate intensity level, they can mutually inhibit one another. But when both noxious and vibratory inputs are strong, they can excite one another.^26^ In some individuals, strong intensities of both stimuli can increase pain intensity.^24^ According to Melzack and Wall,^10^ the experience of pain can be reduced by activation of nerve fibres in the skin (A-β fibres, smaller fibres) that conduct non-noxious stimuli. When moderately stimulated, this can prevent pain signals being transmitted to the central nervous system via A-δ or C fibres (larger fibres). As tactile vibrations are non-noxious, they may reduce pain as they activate A-β fibres, which prevent pain signals being transmitted to the central nervous system via A-δ or C fibres.^27^ Several studies have demonstrated vibration-induced analgesia.^28–30^ For example Hollins et al.^25^ asked participants to rate pain from force applied to the finger with our without vibrations applied to the hand. Participants tolerated more pain with vibrotactile stimulation. Researchers have also shown that combined music and vibration helps those with chronic pain conditions.^31,32^

Although tactile stimulation can affect early sensory processing of pain signals, Melzack and Wall^10^ further proposed that activity in the central nervous system related to factors such as attention, emotion and memory can affect the transmission of pain signals via descending efferent nerves. Thus, attention to pain signals is an important component of the perceived pain intensity. This proposal can be related to two recent accounts that can explain the attenuation of perceived pain intensity by combined auditory and tactile presentation of music.^33–35^ In *attentional-capacity* models, noxious stimulation compete with other sensory stimulation (e.g. sound, touch or both) for limited attentional resources in the central nervous system. Factors like task demands (e.g. difficulty or working-memory load) may recruit limited resources so that people can attend to and carry out the task at hand, leaving fewer resources to process incoming pain signals. People may therefore report lower perceived pain intensity when listening *and* feeling songs because they are allocating most of the attentional resources to the songs rather than the pain signals. By comparison in *attentional-bias* models, psychological factors such as listeners’ emotional or arousal state induced by music may bias attention away from the incoming pain signals towards the music. In this case, people may report lower perceived pain intensity when listening *and* feeling songs because the auditory and tactile information combine to enhance the songs’ affective content and bias people to attend to the songs rather than the pain signals. To summarize, tactile stimulation presented synchronously with music could compete with pain signals (*attentional-capacity* models), or it can modulate listeners’ arousal state to bias attention away from the pain signals (*attentional-bias* models).

To tease apart the *attentional-capacity* and *attentional-bias* models, we measured people’s pain perception whilst they were presented with songs they liked (positive affect) or disliked (negative affect) with or without synchronous tactile stimulation. In the current study, listeners selected a song they liked and one they disliked. We extracted low-frequency amplitude modulations from each song and presented them as vibrations to the wrist. Following procedures established in previous studies,^35,36^ we incrementally applied force to listeners’ fingertip whilst they were presented with their selected song until they reported moderate perceived pain intensity. We compared the force for the *same* perceived moderate pain intensity for each song preference in three stimulation conditions: when a song was only heard (*auditory-only*), only felt (*tactile-only*) or synchronously heard *and* felt (*auditory-tactile*). We hypothesized that the addition of touch enhances the emotion of the preferred song and therefore bias attention away from pain signals (*attentional-bias* models), as we do not expect attentional-capacity differences between listening to the *liked* and *disliked* songs (*attentional-capacity* models). Based on previous studies with chronic pain conditions,^31,32,37–40^ we therefore predicted that presenting listeners with songs *and* synchronous tactile information about the low-frequency amplitude modulations of those songs will attenuate perceived pain intensity. In particular, we predicted that pain attenuation will be largest when listeners can hear *and* feel their preferred song (i.e. the song they like).

## Methods

### Participants

A total of 34 participants (*M* = 20.5 years, *SD* = 1.6 years) were recruited (24 females/10 males). They participated either for course credit or voluntarily, and provided written informed consent. Participants were excluded from the study if they self-reported any medical conditions, injuries or medication (e.g. opioids and anti-depressants) which can influence pain perception in the experimental procedure. This study was approved by the university ethics committee in accordance with the Helsinki Declaration of 1975, as revised in 1983 (Approval reference: 1401_2/15624).

### Materials

#### Force pain induction

**Figure 1.**
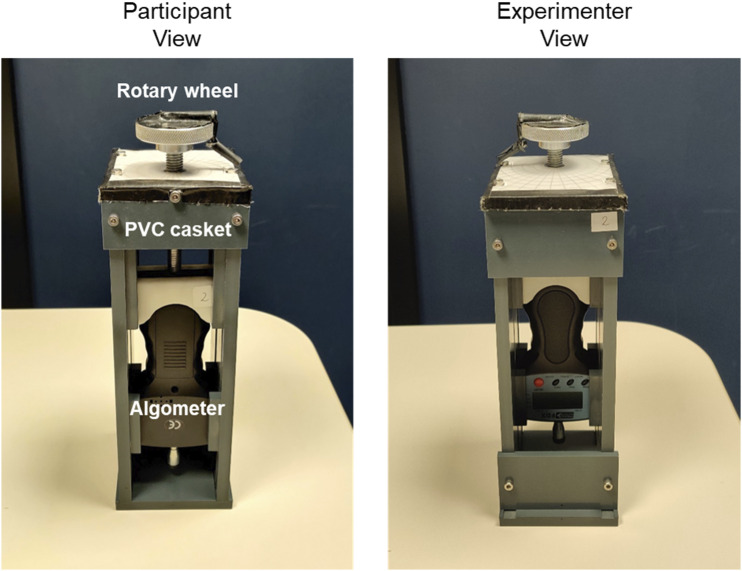
The force induction device from the participant’s (left) and experimenter’s (right) perspective. See also.^35,36^

Figure 1 illustrates the device we used to apply force to the fingertip to induce different pain intensities. Force was measured in Newton (N). This device has been described in detail in Vuong et al.^35^ (see also^36^). Briefly, it consists of a digital algometer (FPX Force 50; model FPX50; resolution: 250 x 0.5 N; Wagner Instruments, Inc.) held in a PVC casket. On the top of the device there is a rotary wheel with markings every 15°. For pain induction, participants placed their hand palm-side down with their fingertip under the algometer’s rubber tip (1 cm diameter). The tip was lowered until it rested on the fingertip and then the algometer was tared; this was the designated rest position (0 N). The experimenter incrementally applied force to the fingertip at a constant rate by rotating the wheel 30° every 1.5 s, using a digital metronome to maintain this rate (tempo = 40 beats/min). Participants were not informed of this constant rotation rate. The experimenter stopped rotating the wheel when participants verbally indicated that a target pain intensity was reached and the Newton value was manually recorded.

The experimenter sat opposite and to the left of the participant throughout the experiment so that the experimenter was not directly in front of participants. The pain-induction device was placed between them at a comfortable distance for the participant. The experimenter manually recorded the force measurement from the algometer’s digital display. Both the measurements and digital display were only visible to the experimenter. The experimenter also wore headphones connected to the metronome to ensure that participants could not hear the metronome’s beat.

#### Songs and auditory stimulation

Twenty songs were used in the current study. The songs were divided into two lists of 10 songs based on a survey by an independent group of respondents. The survey measured arousal and valence ratings for these songs based on their title and artist(s). The songs in List 1 were rated more arousing and more positive than those in List 2. In addition to the human ratings of song titles, we also measured acoustic properties of the songs associated with their affective content. The songs were saved as MP3 files on a Windows 10 laptop. They were played using the VLC software through over-ear headphones (Sennheiser HD380 Pro Headphones) at 40% of the maximum volume. Participants wore the headphones throughout the experiment, with the songs muted (i.e. volume set to 0) during baseline and *tactile-only* trials. The songs and details of the survey and acoustic measurements are provided in the supplementary information (see Table S1).

#### Tactile stimulation

The low-frequency amplitude modulations were extracted from a song and presented to participants using a commercial Basslet watch (Lofelt, Inc.). The watch consists of a wide-band voice coil actuator that band-pass filtered the auditory signal between 45 and 250 Hz. This enabled it to deliver a high-definition tactile experience within the tactile sensitivity range. The actuator was driven by the auditory signal from the laptop in real-time via Bluetooth, and it was set to its maximum vibration strength for this study. The tactile and force stimulation was applied to the same hand, with the actuator worn on the dorsal surface of the wrist (kept in place by the watch straps) approximately 20–25 cm from the fingertip. Participants wore the Basslet watch throughout the experiment, with tactile stimulation turned off during baseline and *auditory-only* trials.

### Design

The experiment used a 2 × 3 within-subjects factorial design. The first factor was the song preference based on the participants’ selection which had two levels: *liked* and *disliked*. The second factor was the stimulation condition which had three levels: *auditory-only*, *tactile-only* and *auditory-tactile*. The dependent variable was the amount of force applied to the fingertip (in N) leading to moderate pain intensity.

### Procedure

Participants were tested individually in a small quiet room by an experimenter. Prior to starting the experiment proper, they were shown the song title and artist(s) from both lists on a sheet of paper. Each participant was then asked to explicitly select one song they *liked* from List 1 and one song they *disliked* from List 2. This manipulation helped to enhance the positive affect for the *liked* song and the negative affect for the *disliked* song. After the song selection and before starting the experiment, participants put on the headphones and watch. They also wore a blindfold so that they could not see their middle fingertip under the algometer’s rubber tip or the experimenter rotating the wheel.

There were two experimental blocks. On each block, there was a baseline phase followed by a stimulation phase. During the baseline phase, we measured the force leading to three subjective pain intensity levels: threshold, moderate and tolerance. The purpose of the baseline phase was to allow the participant to experience the range of pain intensities and to become familiar with moderate pain. The experimenter described each of these levels on a numeric scale from 1 to 10, with threshold being 1 (noticeable level of discomfort), moderate being 7 and tolerance being 10 (maximum pain tolerable). Previous studies have also used this numeric value for moderate pain.^37^ For each intensity level, the experimenter incrementally applied force to the fingertip from 0 N (no force) until participants verbally indicated that the target intensity level was experienced (1 trial per intensity level per block). The three baseline intensity levels were tested in the order threshold, moderate and tolerance on each block. After each baseline trial, a 1-min break was provided to allow participants to rest their finger. The experimenter also reset the algometer to the rest position and manually recorded the force measurement. There was *no* auditory or tactile stimulation during the baseline phase even though participants continued to wear the headphones and watch.

During the stimulation phase, we measured the force leading to moderate pain for each of the six experimental conditions. Participants were presented their *liked* and *disliked* song from its beginning for each stimulation condition (*auditory-only*, *tactile-only* and *auditory-tactile*) for a total of six trials per block. On each trial, the experimenter incrementally applied force to the fingertip from 0 N until participants verbally indicated when they experienced moderate pain intensity (7 out of 10). The six experimental trials were presented in a random order for each participant on each block. After each stimulation trial, a 1-min break was provided to allow participants to rest their finger. The experimenter also reset the algometer to the rest position and manually recorded the force measurement.

Across both blocks, there were two force measurements for the three baseline pain intensity levels and two force measurements for the six experimental conditions. The left or right hand was used on each block, with hand order counterbalanced across participants. A 2-min rest period was given after Block 1. There were three experimenters (the first three authors; two males and one female) and they each tested roughly an equal number of participants. Details of the songs selected by the participants are provided in the supplementary information (see Table S2). An excel file with demographic data, force measurements and selected songs for each participant is available upon request to the corresponding author.

### Data Analysis

We averaged force measurements across the two blocks for the three baseline pain intensity levels and the six experimental conditions. The mean force measurements from the experimental conditions were submitted to a 2 × 3 repeated-measures analysis of variance (ANOVA) with song preference and stimulation condition as within-subjects variables. For post-hoc comparisons, we used Bonferroni correction and report corrected *p*-values (i.e. *p*-value x number of possible comparisons). A significance level of α = .05 was adopted for all statistical analyses reported.

## Results

**Figure 2.**
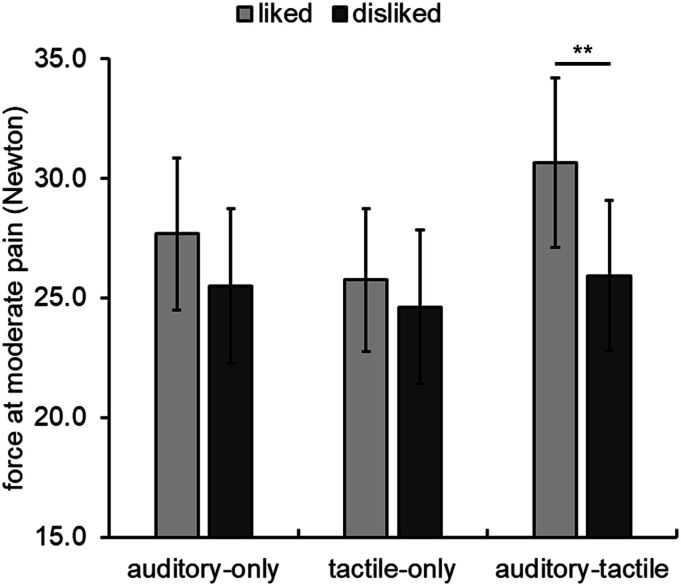
Mean force at moderate pain as a function of stimulation condition and song preference. Error bars represent standard error of the mean. ** *p* = .0006.

Tables 1 and 2 present mean force (in Newton) in the baseline and stimulation phases of the experiment, respectively. Figure 2 illustrates the mean force in the stimulation phase. The values represent the mean force applied to the fingertip when participants reported experiencing a target subjective pain intensity (e.g. subjective moderate pain intensity). Thus, when comparing two conditions, a *higher* force measurement can be interpreted as an attenuation in perceived pain intensity (i.e. more force is needed for the *same* perceived pain intensity).

**Table 1.** Mean and standard error (in Newton) for force as a function of baseline perceived pain intensity level.

	Threshold	Moderate	Tolerance
Mean	5.58	23.19	48.88
SE.	0.82	2.48	4.92

**Table 2.** Mean and standard error (Newton) for force at moderate pain as a function of stimulation condition and song preference. %Att is the percentage attenuation in perceived pain intensity relative to baseline moderate force measurement.

	Auditory-only		Tactile-only		Auditory-tactile	
	Liked	Disliked	Liked	Disliked	Liked	Disliked
Mean	27.68^a^	25.50	25.76	24.63	30.67^a,b^	25.93
SE.	3.17	3.24	3.00	3.23	3.53	3.14
%Att	8.82	4.72	5.24	3.00	13.88	5.58

^a^significantly different from baseline moderate force measurement.

^b^significant difference between liked and disliked songs.

For the stimulation phase, the ANOVA indicated that there were significant main effects of song preference, *F* (1,33) = 19.04, *p* < .001, $\eta_{p}^{2}$ = .366; and stimulation condition, *F* (2,66) = 10.61, *p* < .001, $\eta_{p}^{2}$ = .243. Force at moderate pain was higher for the *liked* song (*M* = 28.04 *N*, *SE* = 3.18 N) compared to the *disliked* song (*M* = 25.35 *N*, *SE* = 3.18 N). Post-hoc paired-samples *t*-tests (two-tailed) were carried out to determine differences in force at moderate pain between the three stimulation conditions (three possible comparisons). The *auditory-tactile* condition elicited larger force at moderate pain (*M* = 28.30 *N*, *SE* = 3.30 N) compared to only the *tactile-only* condition (*M* = 25.19 *N*, *SE* = 3.09 N), *t* (33) = 5.02, *p* = .0002. No other pairwise comparisons were significant after Bonferroni correction.

The main effects were qualified by a significant interaction between song preference and stimulation condition, *F* (2,66) = 4.02, *p* = .023, $\eta_{p}^{2}$ = .109. We therefore conducted post-hoc paired-samples *t*-tests to compare force measurements for the *liked* and *disliked* songs separately for each stimulation condition (15 possible comparisons). There was no effect of song preference for the *auditory-only* and *tactile-only* conditions. By comparison, force at moderate pain for the *liked* song was larger than for the *disliked* song in the *auditory-tactile* condition, *t* (33) = 4.72, *p* = .0006 (see Table 1 and Figure 1). Lastly, we compared force at moderate pain for the *liked* song in the *auditory-tactile* condition to the remaining five experimental conditions (five possible comparisons). The force measurement was highest in this condition compared to all the other conditions (*t*s > 4.71, *p*s < .001) except for the *liked* song in the *auditory-only* condition.

For comparison to previous work, we conducted exploratory *t*-tests to determine differences in force at moderate pain between baseline and experimental conditions (nine possible comparisons). Recall that force measurements at baseline was collected before the stimulation phase on each block thus precluding ANOVAs including the baseline. In line with previous work,^6^ force at moderate pain was significantly higher in the *auditory-only* condition compared to the baseline condition only for the *liked* song, *t* (33) = 4.13, *p* = .001. Similarly, force at moderate pain was significantly higher in the *auditory-tactile* condition compared to baseline only for the *liked* song, *t* (33) = 4.25, *p* = .0009. Force measurements was also significantly higher in the *auditory-tactile* condition compared to baseline, *t* (33) = 3.41, *p* = .03. Tactile stimulation by itself, however, did not affect force measurements relative to baseline, contrary to what was previously found.^24,25^ All other comparisons were not significant after Bonferroni correction. Following previous work,^35^ we also calculated the percentage pain attenuation using the mean force for each of the six experimental conditions (see Table 2). This attenuation varied from approximately 3–14%, with numerically larger attenuation for the *liked* compared to the *disliked* song in all conditions.

## Discussion

The current study investigated whether the addition of tactile stimulation can affect music-induced analgesia. We incrementally applied force to participants’ fingertip, and measured the force (in Newton) at which they perceived moderate pain intensity. This procedure allowed us to directly measure pain perception in different conditions (see also^35^). Consistent with our predictions, presenting listeners with music *and* synchronous tactile information about the low-frequency amplitude modulations of that music attenuated participants’ perceived pain intensity. Listeners had significantly higher force measurements for the same perceived moderate pain intensity for their *liked* compared to *disliked* song, and in the *auditory-tactile* compared to the *tactile-only* stimulation condition. These main effects were qualified by how song preference interacted with stimulation condition: Listeners had significantly higher force measurements for their *liked* compared to *disliked* song only in the *auditory-tactile* condition; and they had higher force measurements for their *liked* song with *auditory-tactile* stimulation compared to the other remaining conditions except for the *liked* song with *auditory-only* stimulation.

Our findings demonstrate that presenting listeners’ preferred song synchronously with its corresponding low-frequency vibrations is more effective at attenuating pain perception than presenting the preferred song or the vibroacoustics associated with that song in isolation. The significant interaction between song preference and stimulation condition allows us to further understand the relative contributions of different factors to MIA. Firstly, the affective content of music by itself may not be sufficient to attenuate pain perception because the *liked* songs were generally rated to be more arousing and positive than the *disliked* songs^20^ (see supplementary Table S1). Secondly, listeners’ individual music preferences or their ability to control the music selection may also not be sufficient by themselves to attenuate pain perception because listeners in our study selected a song from each list.^23,41,42^ Thirdly, tactile stimulation by itself may not be sufficient to attenuate pain perception as previously reported.^24,25^ Rather, these different factors may combine synergistically to attenuate the perceived pain intensity from noxious force on the fingertip.

Taken together with previous work, our results help to differentiate the attentional-capacity and attentional-bias models of pain attenuation.^33–35^ In particular, attentional-capacity models, in which pain signals compete with other sensory stimulation for limited attentional resources, are less able to account for the findings here. If this was the case, we would not expect differences in force measurements for the same perceived moderate pain intensity between the *liked* song and the *disliked* song in the *auditory-tactile* stimulation, since the sensory stimulation would be similar for both song preferences. Rather our current study supports attentional-bias models of pain perception in which psychological factors such as listeners’ preference for a song may bias attention away from pain signals. We speculate that synchronous tactile stimulation may enhance the affective content of the *liked* song but not the *disliked* song which, in turn, can bias listeners away from pain signals when hearing and feeling their *liked* song. This condition attenuated perceived pain intensity by approximately 14% relative to baseline.

The current study aimed to test whether auditory-tactile stimulation can enhance pain attenuation so we did not explicitly measure how tactile stimulation affected participants’ affective and emotional state, nor did we measure how it affected participants’ mood. Based on previous work, we make some speculations here. Firstly, the findings suggest that synchronous tactile stimulation can enhance the affective content in music. This interpretation is consistent with findings from Branje et al.^12^ who found that the addition of tactile stimulation to music induced stronger emotional responses in film music (see also^13^). Secondly, the tactile stimulation can enhance the music experience by inducing a greater sense of arousal (deeper experience), which was a finding also present in Branje et al.^12^ Thus, in the *liked* song with *auditory-tactile* stimulation, listeners may better experience pleasant emotions associated with their preferred song. These enhanced pleasant emotions may activate descending pain-modulatory mechanisms via dopamine and endogenous opioids to inhibit pain transmission.^4,9,43^ Both possibilities can be tested in future studies by correlating participants’ affective and emotional ratings of the songs in different stimulation conditions and the magnitude of pain attenuation in those conditions. Future studies can also incorporate objective physiological and neural measurements of arousal and mood (e.g. heart rate, skin conductance, EEG activity) to assess whether they corroborate the force measurements.

The finding that tactile stimulation alone was not significantly different than baseline contrasts with previous studies which demonstrated pain attenuation by low-frequency tactile vibrations.^24,28–30^ There may be at least two reasons for the differences in results. Firstly and perhaps critically, previous studies used a single low-frequency vibration between 12 and 80 Hz. By comparison, the Basslet watch extracted a wider low-frequency bandwidth which included frequencies between 45 and 250 Hz. In the absence of sounds, a single-frequency vibration would also be more predictable (i.e. tactile stimulation at a regular interval) than the wide-frequency vibration extracted from a song. This predictability may affect attention to pain signals which may be unexpected; thus, single-frequency vibrations may be more likely to attenuate pain perception. Secondly, the amplitude of tactile stimulation can interact with the frequency of stimulation. For example Hollins et al.^24^ found that participants rated force applied to the fingertip as more painful when high-amplitude 80-Hz vibrations was applied compared to either low-amplitude 80-Hz vibrations or high-amplitude vibrations at lower frequencies (12 or 50 Hz). In our study, some participants may not be able to experience pain modulation from tactile stimulation alone as the strength of the Basslet watch was set to its maximum amplitude. Thus, when force was incrementally increased, both perceived pain and vibration were intense, possibly nullifying the pain-modulating effect of vibrations. This is because when both noxious and non-noxious tactile inputs are at a strong intensity, they excite one another.^11,26^ That said, the wide-frequency strong tactile stimulation combined with preferred music can attenuate perceived pain intensity. In future studies, it may be important to systematically measure different vibration parameters (e.g. amplitude, frequency bandwidth and predictability) to modulate pain experiences under different music conditions.

There are also limitations to consider for this study. Firstly, we assumed that tactile stimulation by itself can induce emotion in listeners. This assumption was based on a study by Sharp et al.^44^ who demonstrated that both musicians and non-musicians were able to recognize emotions via tactile stimulation above chance for music that induced emotions of happiness and fear. It may be worthwhile in future studies to measure the characteristics of the tactile stimulation extracted from music and determine which characteristic may modulate pain experience as done for the acoustic properties of music.^22,45–47^ A second potential limitation is that we did not systematically control general attention. For example the addition of tactile stimulation can increase listeners’ attentive state. Although general attention may partly account for differences in force at moderate pain between the *auditory-tactile* compared to the single sensory conditions, it does not account for the interaction with song preference. A final potential limitation is that we selected music with lyrics and the songs were presented from their beginning on each trial. Listeners may consequently use the lyrics to determine when moderate pain was reached rather than basing their decision on the perceived pain intensity at their fingertip. We did not ask participants what strategies they used during the experiment but we note that this lyric strategy can be used for both the *liked* and *disliked* song. However, we found that force measurements at moderate pain were only significantly different from baseline for the *liked* song in the *auditory-only* and *auditory-tactile* stimulation conditions (see Table 2). It would be interesting in future studies to compare the role that lyrics may have in MIA, as most previous studies used music without lyrics.

We acknowledge that different participants were presented with different experimental songs for the *liked* and *disliked* conditions, which may limit the generality of our results. This methodological limitation is a general issue for MIA research. We also note that the incremental pain induction procedure is not automated; thus, there may be some bias introduced by the different experimenters. All three experimenters were well-practiced on the incremental pain induction procedure. Moreover, we used a constant rotation rate (30° every 1.5 s) maintained by a metronome (see also^35^). These steps help to reduce any experimenter bias influencing the results.

To summarize, we demonstrated that pain attenuation was highest when listeners could *feel and hear the song they liked*. Vibroacoustics is increasingly used to enhance the affective and emotional experience of music for listeners.^12,13,44^ In line with this, providing tactile stimulation may enhance the positive emotions of preferred music and increase the likelihood that top-down affective and motivation mechanisms would attenuate negative aspects of the pain experience such as its intensity or unpleasantness. In the growing search for non-pharmacological interventions for acute and chronic pain in real-world situations (e.g. at the dentist^48^), combined tactile stimulation with preferred music listening has the potential to be a simple yet powerful tool to attenuate negative pain experiences.

## Supplemental Material

Supplemental Material - Feeling the music: The feel and sound of songs attenuate pain Click here for additional data file. Supplemental material for Feeling the music: The feel and sound of songs attenuate pain by Dhillon Lad, Alex Wilkins, Emma Johnstone and Quoc C Vuong in British Journal of Pain
