# Supplementary material for: Feeling the music: The feel and sound of songs attenuate pain
Source: Br J Pain. 2022 May 3;16(5):518–27. doi: 10.1177/20494637221097786 (PMC9644099; doi:10.1177/20494637221097786)
Supplement: Supplemental Material - Feeling the music: The feel and sound of songs attenuate pain [file sj-pdf-1-bjp-10.1177_20494637221097786.pdf]

Supplementary Information

Affective content of songs

A Qualtrics survey was created and shared online to measure the affective content of 20 songs with lyrics (see Table S1). The 20 songs were chosen by the experimenters through informal consultation with their peer group to ensure that respondents for the survey were familiar with the songs, since only the song title and artist(s) were provided. The song titles and artists were presented in random order to each respondent. Note that the song was not necessarily performed by the original artists. Respondents were asked to rate the arousal and the valence of each song on a 5-point scale only if they were familiar with the song and artist. For arousal ratings, the scale varied from 1 (Least Arousing) to 5 (Most Arousing). For valence ratings, the scale varied from 1 (Negative Valence) to 5 (Positive Valence). Arousal and valence was defined at the beginning of the survey based on the International Affective Picture System.[1] In total there were 75 respondents but only 58 completed the survey. Of these there were 24 males, 33 females and 1 undisclosed, with a mean age of 23.8 years and a standard deviation of 9.2 years. From the responses, for each song a mean for arousal and a mean for valence was calculated. List 1 included songs which respondents rated as more arousing and more positive. List 2 included songs which respondents rated as less arousing and more negative.

To complement these ratings based only on song title and artist(s), we also measured acoustic attributes that are known to be associated with the affective content of music.[2-4] First, we used Spotify's auditory analysis to calculate the songs' acoustic energy (arousal) and valence.[5] Second, we use the MIRtoolbox[6] for Matlab to compute the average rhythm (tempo, in beats per min [bpm]), pitch (in Hz) and timbre (brightness and roughness [unitless]) for each song. After Beferroni correction, only arousal and valence ratings differed between *liked* and *disliked* songs, $t(18) = 4.22$, $p = .005$, and $t(18) = 4.47$, $p = .003$, respectively. We further note that for Spotify's energy acoustic measurement, $t(18) = 2.49$, $p = .023$ (uncorrected), which was not significant after Bonferroni correction.

Table S1.

Different measures of affective content for liked and disliked songs. Note that Spotify Energy is a measure of Arousal.
**significant difference between liked and disliked song*

Song & Artist(s)	Arousal*	Valence*	Spotify Energy	Spotify Valence	Rhythm (tempo, bpm)	Pitch (Hz)	Timbre (brightness)	Timbre (roughness)
**List 1 (*Liked*)**								
Come On Eileen – *Dexys Midnight Runners*	2.78	2.86	0.66	0.78	116.59	431.96	0.54	1799.28
Dancing Queen – *ABBA*	3.33	3.52	0.87	0.75	107.82	625.52	0.56	188.23
Dancing in the Moonlight – *Toploader*	3.07	3.29	0.62	0.19	103.15	549.45	0.47	1263.52
Mr Brightside – *The Killers*	3.07	3.10	0.93	0.24	140.68	546.42	0.56	4910.33
Shotgun – *George Ezra*	2.48	2.64	0.74	0.75	116.77	420.78	0.45	1730.85
One Dance – *Drake*	2.79	2.83	0.63	0.37	103.96	436.60	0.38	469.37
Old Town Road – *Lil Nas X*	2.60	2.71	0.53	0.51	121.59	507.36	0.41	1546.98
YMCA – *Village People*	2.90	3.34	0.97	0.73	126.89	544.93	0.56	79.18
Uptown Funk – *Mark Ronson & Bruno Mars*	3.17	3.22	0.61	0.93	114.91	645.81	0.55	1198.36
Shape of You – *Ed Sheeran*	2.76	3.00	0.65	0.93	127.62	382.30	0.40	1583.57
Mean (*SD*)	2.89 (0.26)	3.05 (0.29)	0.72 (0.15)	0.62 (0.27)	118.00 (11.71)	509.01 (89.31)	0.49 (0.07)	1476.97 (1363.24)
**List 2 (*Disliked*)**								
Senorita – *Shawn Mendes & Camillo Cabella*	2.40	2.48	0.54	0.75	116.71	464.68	0.45	1464.87
Hey There Delilah – *Plain White T's*	1.93	2.00	0.29	0.30	115.85	285.62	0.21	391.49
Thriller – *Michael Jackson*	3.05	3.17	0.89	0.72	118.74	577.65	0.49	239.64
Shake It Off – *Taylor Swift*	2.47	2.71	0.80	0.94	127.32	707.89	0.55	2055.64
Call Me Maybe – *Carly Rae Jepsen*	2.34	2.55	0.58	0.66	119.05	550.98	0.54	315.09
I Will Always Love You – *Whitney Houston*	2.55	2.29	0.21	0.11	126.60	530.14	0.35	31.44
Dancing On My Own – *Calum Scott*	2.09	1.83	0.17	0.24	113.89	636.52	0.31	103.66
You're Beautiful – *James Blunt*	2.24	2.31	0.48	0.45	148.95	399.71	0.39	364.65
Bleeding Love – *Leona Lewis*	2.12	1.81	0.65	0.20	118.00	582.27	0.44	744.46
Someone Like You – *Adele*	2.34	1.95	0.32	0.29	118.49	397.66	0.34	507.36
Mean (*SD*)	2.35 (0.31)	2.31 (0.43)	0.47 (0.28)	0.13 (0.07)	122.36 (10.29)	513.31 (126.55)	0.41 (0.11)	621.83 (647.31)

Mean arousal and valence ratings for selected songs

In the main study, a different group of participants (N = 34) selected a *liked* song from List 1 and a *disliked* song from List 2. We therefore calculated the mean ratings for each affective dimension for the selected songs. Table S2 presents the mean arousal and valence ratings for the *liked* and *disliked* songs selected by participants in the main study. Due to loss of some data, the selected *liked* and *disliked* songs were available for only 20 out of 34 participants.

Table S2.

Mean (standard deviation) arousal and valence ratings for the selected liked and disliked songs. Note that the descriptive statistics are based on N = 20 participants.

	liked songs	*disliked* songs
Arousal	2.86 (0.29)	2.39 (0.28)
Valence	2.98 (0.31)	2.47 (0.35)

Supplementary References

1. Bradley MM and Lang PJ. The International Affective Picture System (IAPS) in the study of emotion and attention. In: Coan JA and Allen JJB (eds) Handbook of emotion elicitation and assessment. Oxford: Oxford University Press, 2007, pp. 29-46.

2. Eerola T, Lartillot O and Toiviainen P. Prediction of multidimensional emotional ratings in music from audio using multivariate regression models. In: 10th International Society for Music Information Retrieval Conference, 2009, pp. 621-626.

3. Howlin C and Rooney B. Patients choose music with high energy, danceability, and lyrics in analgesic music listening. *Psychol Music*, 2021; 49: 931-944.

4. Knox D, Beveridge S, Mitchell LA, et al. Acoustic analysis and mood classification of pain relieving music. *J Acoust Soc Am* 2011; 130: 1673-1682.

5. Spotify. Get audio features for a track, https://developer.spotify.com/documentation/web-api/reference/tracks/get-audio-features/ (2019, accessed November 1, 2021).

6. Lartillot O and Toiviainen P. A Matlab toolbox for musical feature extraction from audio. In: Proceedings of the 10th International Conference on Digital Audio Effects (DAFx-07), 2007, pp. 1-8.
